# Notes and News

**Published:** 1896-03-28

**Authors:** 


### Notes and News.

(Collected and Communicated.)

HOSPITAL MEETINGS.

The Annual Court of the Royal Orthopædic Hospital, Hanover Square, will be held at the Hospital on Tuesday, March 31st, at 5 o'clock.

His Grace the Duke of Fife will preside at the 45th Anniversary Festival of the City Orthopædic Hospital on Wednesday, April 29th, at the Holborn Restaurant.

Mrs. Anna Aspinwall has bequeathed 20,000 dols. for the establishment of a convalescent home for children, and 3,000,000 dols. in trust to the hospital of the Episcopal Church of Philadelphia.

The necessity for full inquiry in every case before relief is given is illustrated by the impositions which have been practised recently by a mendicant upon the charitable public. A man by the name of Robert Carr has been obtaining money from various sources to assist him in various projects put forth in begging letters. He was finally exposed by stating that he was a patient at the Cancer Hospital, Brompton. Inquiry proved that he had never entered the institution. Imprisonment for one year, with hard labour, was awarded to him.

It seems likely that the Belgravia Hospital for Children will change its quarters. A meeting to consider this scheme was held at Grosvenor House last week. The proposal was to remove it to South London. Mr. Denton went so far as to say the question was one as to whether the hospital should "perish at Pimlico or prosper at Kennington." An amendment was ultimately carried which provided for the reconstituting of the hospital in its present neighbourhood. It was mentioned during the meeting that the Duke of Westminster had expressed his willingness to offer a site in Ebury Square. As a site is required which will permit of the erection of an institution containing fifty beds, it is doubtful whether the one offered will be large enough.

At the Hotel Metropole, on Thursday, March 19th, the thirty-second anniversary banquet of the Home for Little Boys took place, under the presidency of Sir George Newnes. There were some 200 ladies and gentlemen present. Subscriptions, &c., were announced amounting to £1,500.

The National Hospital for Consumption for Ireland, the plans of which we published on June 8th, 1895, was formally opened by the Marchioness of Zetland on the 19th inst. The building, which is a very fine one, is situated at Newcastle, Newtown Mountkennedy, co. Wicklow. The building is divided into three blocks. The design is in Old English style, and the central block is connected with the others by conservatories. The building stands on an eminence, and the patients' bed-rooms open on to verandahs, which command fine views. The committee will be glad to receive presents of pictures, books, and plants for the institution. The originator of the institution was Miss Florence Wynne, who worked very hard to establish the scheme, and acted as honorary secretary for some time. Later the late Duchess of Leinster, the Marchioness of Zetland, and many other influential ladies took up the cause.

There is no provision for medical cases in the Chesterfield Hospital. The district is one in which accidents and injuries are frequent, and the accommodation provided is none too great for surgical requirements. Nevertheless, it has lately dawned upon the Chesterfield mind that the inhabitants are not wholly medically exempt from the need of hospital aid, and a proposal has been discussed by the authorities to admit medical patients and provide extra accommodation for them if necessary. The question was brought up at the annual meeting, and the discussion that followed was carried on in a very discreditable manner by some of the speakers. The arguments both for and against the proposition were strikingly lacking in common sense, and no consensus of opinion was apparent until the motion to provide medical accommodation was put to the vote. It was then rejected by a large majority, and so the inhabitants of Chesterfield must, as before, be satisfied with such care in illness as they can obtain in their own homes, however unsuitable.

We have just received the statement of accounts of St. Bartholomew's Hospital for the year ended December 31st, 1895. We observe with satisfaction that for the first time they have been prepared upon the uniform system, a circumstance which reflects the greatest credit upon Mr. Cross, Sir Trevor Lawrence (the treasurer), and all who have been instrumental in putting this salutary change into execution. It is pleasant to find that, in deciding upon the new form of accounts, St. Bartholomew's Hospital has gone one better than the uniform system by stating in separate columns the amount expended under each head on provisions (*a*) for the patients, and (*b*) for the nursing staff. It cannot fail to have caused some trouble to have reconstructed the accounts of St. Bartholomew's Hospital, but the result is eminently satisfactory. We hope that St. Thomas's Hospital, the last to cling to the old and complicated system of the past, will now follow suit, and that the accounts of Guy's Hospital may be made up to December 31st in each year instead of, as at present, to March 25th, a plan which creates many objections and complications which were best obviated.

